# The technique for less infectious and earlier healing of stoma closure wound: negative pressure wound therapy with instillation and dwelling followed by primary closure

**DOI:** 10.1186/s12893-021-01109-2

**Published:** 2021-03-22

**Authors:** Yoshinori Yane, Jin-ichi Hida, Yusuke Makutani, Hokuto Ushijima, Yasumasa Yoshioka, Masayoshi Iwamoto, Toshiaki Wada, Koji Daito, Tadao Tokoro, Kazuki Ueda, Junichiro Kawamura

**Affiliations:** 1 Department of Surgery, Faculty of Medicine, Kindai University, Osaka-sayama, Osaka, Japan; 2 Department of Gastroenterological Surgery, Kindai University Nara Hospital, Ikoma, Nara Japan

**Keywords:** Stoma closure, Surgical site infection, SSI, NPWTi-d, Delayed primary closure, V.A.C. VERAFLO Therapy

## Abstract

**Background:**

Temporary stomas have been widely used to avoid the risk of complications such as anastomotic leakage after colorectal resection. Stoma closure is relatively easy; however, postoperative surgical site infection (SSI) may be a problem. Various methods have been used to reduce the incidence of SSI. We aimed to evaluate a new technique for stoma wound closure.

**Methods:**

We enrolled patients who underwent stoma closure at our hospital between September 2019 and May 2020. We selected patients who lived far from our hospital and had difficulty visiting the hospital regularly and who agreed to undergo this surgical technique. We used negative pressure wound therapy with instillation and dwelling (NPWTi-d) and delayed primary closure for these patients.

**Results:**

Four patients underwent NPWTi-d and delayed primary closure without the occurrence of SSI. The median postoperative hospital stay was 9 days (range: 7–14 days), and the median number of days to confirmation of epithelialization was 11.5 days (range: 10–16 days).

**Conclusion:**

The combined use of NPWTi-d and delayed primary closure for the stoma wound was very effective. This method may be a valuable new technique for wound management after stoma closure.

## Background

The frequency of temporary stoma creation has increased because of improved techniques of sphincter-saving surgery for lower rectal cancer. Stoma closure is a relatively easy operation; however, surgical site infection (SSI) following stoma closure increases the burden on patients, duration of admission, and medical costs [1]. Furthermore, as a serious late complication of stoma closure, SSI may be a risk factor for abdominal incisional hernia formation [2].

The usual method for skin closure at the stoma site is primary suturing; however, the incidence of SSI is reportedly as high as 40% [3]. Therefore, purse-string skin closure has been widely used as a preventive measure [4]. Another method that has been reported is negative pressure wound therapy (NPWT) [5, 6]. In NPWT, a sealed wound dressing attached to a vacuum pump sucks fluid away from the wound and is thought to promote angiogenesis, reduce edema, increase tensile strength, and reduce SSI. However, there are few reports of the use of NPWT for stoma closure, and its efficacy has not been clearly shown. Purse-string skin closure is reportedly very successful for SSI prevention [4]; however, epithelialization of the wound takes a long time [7]. NPWT is very useful for granulation; however, there are cases in which local infection developed [5].

The use of NPWT with instillation and dwelling (NPWTi-d) is gaining interest. NPWTi-d has the benefits of NPWT, with additional automatic cleaning of the wound surface and dissolving of devitalized tissue for removal. It can assist with early, aggressive removal of exudate and be used to lessen the bacterial load. Although NPWTi-d was initially used largely as a last-resort therapy and is currently used in various types of wound, it has not been shown to be effective for use in stoma closure.

We hypothesized that the use of NPWTi-d for stoma wound closure could reduce the risk of infection and improve wound management. However, the time to epithelialization of the wound and consequent burden on patients may still be relatively long. Therefore, in addition to the use of NPWTi-d, we believed that delayed primary closure might reduce the burden of treatment and shorten the postoperative hospital stay, and the number of outpatient visits.

## Methods

### Patients

Between September 2019 and May 2020, we performed 20 stoma closures at our hospital. We selected patients who lived far from our hospital and had difficulty visiting the hospital regularly, who wanted to try this surgical technique.

### Surgical technique

**Fig. 1:**
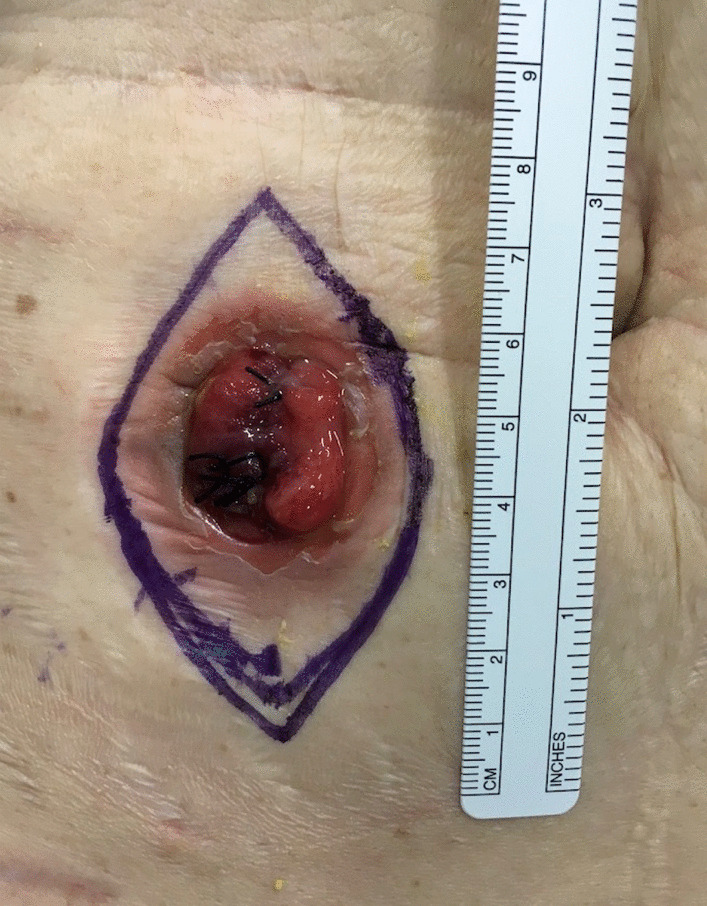
A spindle-shaped skin incision was made in the cranio-caudal direction with a length three times the lateral diameter of the stoma

**Fig. 2:**
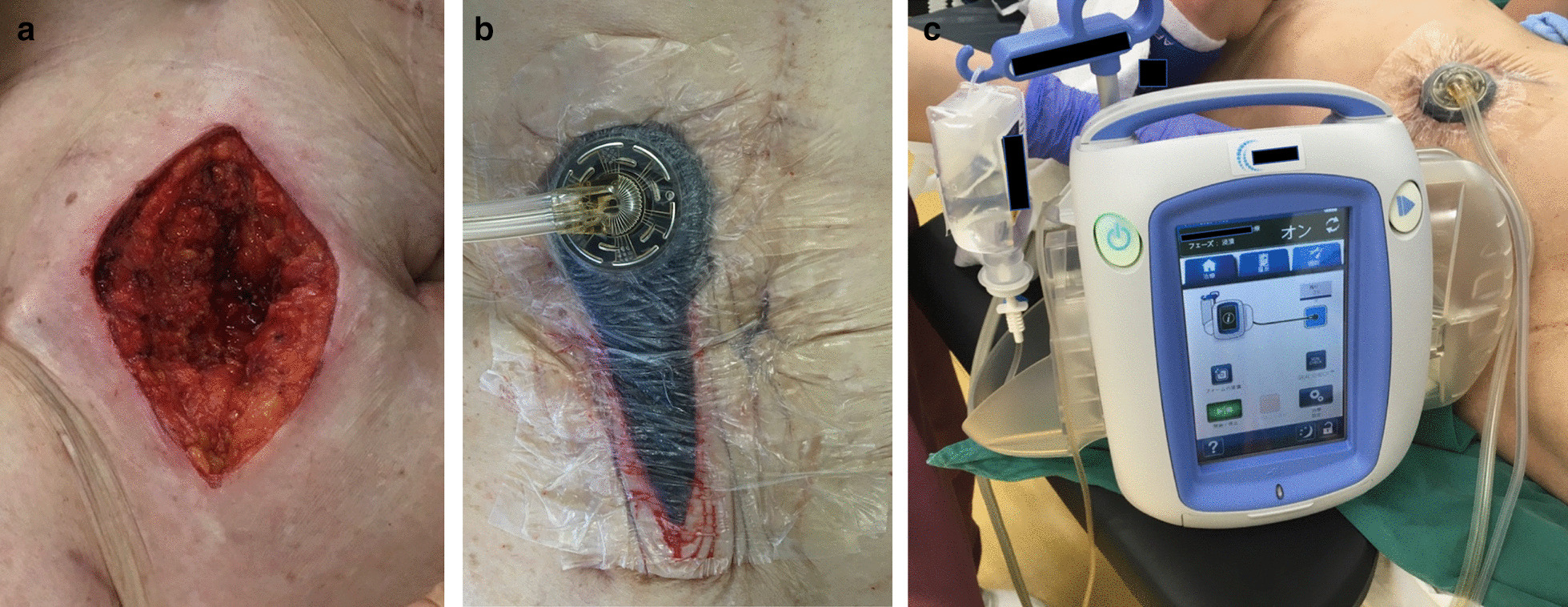
Fascial closure was performed to an interrupted manner with an 0 polydioxanone suture (PDS®). Subcutaneous tissue and skin was not sutured (**a**). V.A.C. VeraFlo™ was attached to the wound (**b**, **c**)

A spindle-shaped skin incision was made in the cranio-caudal direction with a length three times the lateral diameter of the stoma (Fig. 1). The subcutaneous tissue around the stoma was incised, and the adhesions around the small bowel were detached from the abdominal wall. After the small bowel had been mobilized, the segment that had remained outside the abdominal wall was excised. A functional end-to-end anastomosis was then created. Interrupted sutures (0 polydioxanone, PDS®, Ethicon, Cincinnati, Ohio., USA) were used for fascial closure. After confirming hemostasis, the subcutaneous tissue was washed with 1500 ml of physiological saline. The subcutaneous tissue and skin were not sutured, and NPWTi-d (V.A.C. VERAFLO Therapy, KCI, an Acelity Company, San Antonio, Texas) was attached directly to the wound (Fig.2a–c).

### Preoperative and postoperative care

According to our hospital protocol, all patients who underwent stoma closure received mechanical preparation with oral laxatives. Antibiotic prophylaxis, 1.0 g flomoxef sodium (Flumarin®, Shionogi & Co., LTD, Osaka, Japan) was administered 30 min before the incision, and a single additional dose was given 3 h after surgery. The first drink was given on postoperative day 1 (POD1), and the first solid oral intake was POD3.

### NPWTi-d and delayed primary closure

**Fig. 3:**
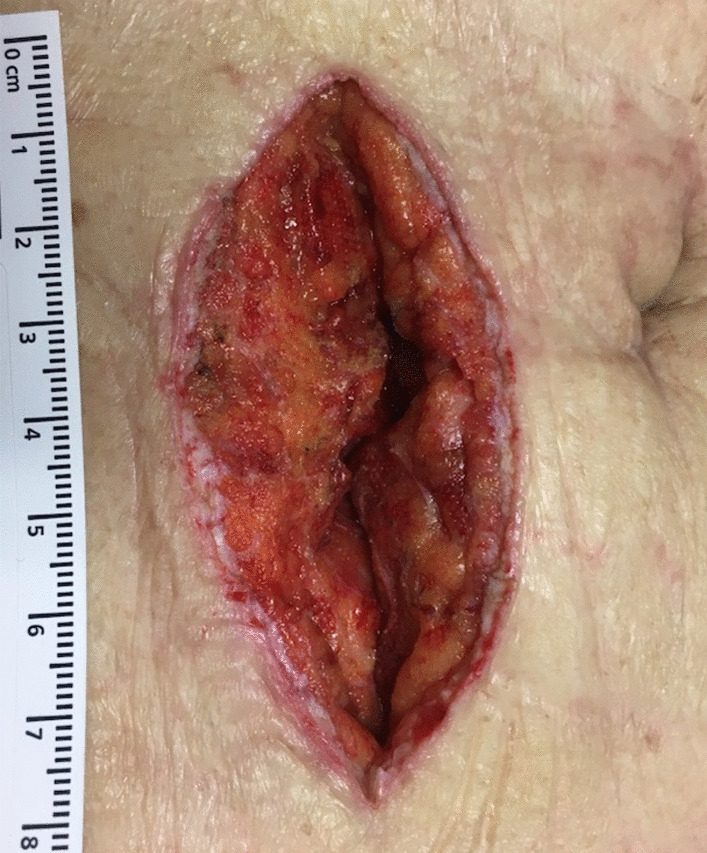
The granulation had grown to the extent that the abdominal rectus muscle fascia and the suture was covered. There was no obstruction of blood flow in the granulation

**Fig. 4:**
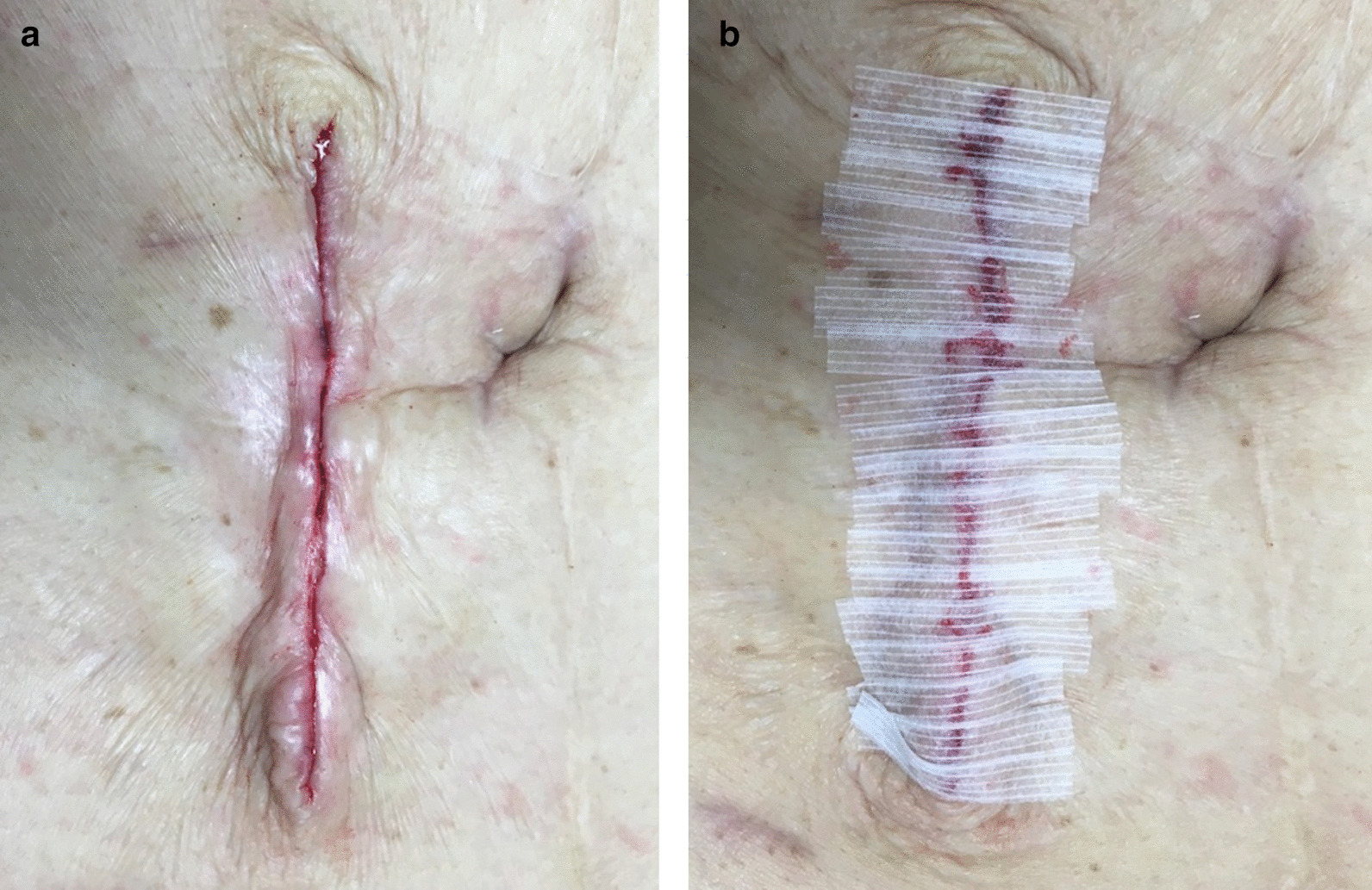
It was performed delayed primary closure by subcuticular suture with 4–0 PDS (**a**). The epidermis was reinforced with 3 M™ Steri-Strip™ Skin Closures (**b**)

Immediately after surgery, NPWTi-d was attached to the wound and treatment was started. The V.A.C. VeraFlo™ settings were set to an instill volume of 2 ml, soak time of 2 min and a V.A.C.® therapy time of 2 h. The target pressure was 75 mmHg, and the intensity was low. The foam was removed at POD 3 or POD 4. If it was confirmed that the granulation had covered the abdominal rectus muscle fascia and the sutures, and there was no obstruction of blood flow in the granulation, delayed primary closure was performed (Fig. 3). The wound was washed with 100 ml of physiological saline, and subcuticular sutures (4–0 PDS) were used (Fig. 4a). The epidermis was reinforced with Steri-Strip™ skin closures (3 M Japan Limited, Tokyo, Japan) to reduce tension on the sutures (Fig. 4b).

## Results

Four patients were treated with NPWTi-d and delayed primary closure for wound management. There were 3 males and 1 female. The median age was 72 years (range: 44–75 years). The median body mass index (BMI) was 20.7 kg/m^2^ (range: 17.6–36.9 kg/m^2^). One patient was obese (BMI > 30 kg/m^2^). One patient (25%) had a history of diabetes mellitus, and one patient (25%) had received chemotherapy. Two patients (50%) were smokers. Three patients (75%) had a low anterior resection, and 1 patient (25%) had a high anterior resection for rectal cancer. All patients had a subsequent ileostomy (Table 1).

**Table 1:** Clinical data on 4 patients undergoing negative pressure wound therapy with instillation and dwelling (NPWTi-d) followed by delayed primary closure: Before stoma closure

Case no	Age range, years	Sex	ASA class	Bmi (kg/m^2^)	Smoking	Comorbidity	Alb	BUN	Cr	HbA1C	Dragnosis	First ope	Diverting stoma	Adjuvant chemotherapy
1	70–79	2	3	17.6	+	None	3.9	16	0.7	5.6	Rectal cancer	Lap-LAR	Ileostomy	−
2	70–79	1	3	17.6	−	DM	3.6	24	0.9	7.1	Rectal cancer	Lap-LAR	Ileostomy	−
3	70–79	1	2	23.5	−	None	3.3	38	2	5.9	Rectal cancer	Lap-LAR	Ileostomy	−
4	40–49	1	2	36.9	+	None	3.9	14	0.8	5.7	Rectal cancer	Lap-LAR	Ileostomy	+

*ASA* American Society of Anesthesiologists, *BMI* body mass index, *Alb* Albumin, *BUN* Blood urea nitrogen, *Cr* Creatinine, *Ope* Operation, *DM* Diabetes mellitus, *Lap-LAR* Laparoscopic low anterior resection, *Lap-HAR* Laparoscopic high anterior resectio

Table 2 presents the details of stoma closure and outcomes. No wound bleeding was observed during NPWTi-d. In all cases, the first foam exchange was performed on POD 3 or 4 and delayed primary closure was performed because granulation had grown enough to cover the abdominal rectus muscle fascia and the sutures.

**Table 2:** Clinical data on 4 patients undergoing negative pressure wound therapy with instillation and dwelling (NPWTi-d) followed by delayed primary closure: After stoma closure

Case no	Stoma closure day after 1 stope	Stoma closure ope. Time (min)	Duration of NPWTi-d	SSI	Postoperative hospital stay (days)	Outpatient visit after discharge (times)	Wound healed (postoperative days)	Post ope. course
1	175	75	3	−	11	0	11	Unremarkable
2	119	102	3	−	14	1	10	SBO
3	150	74	4	−	7	1	16	Unremarkable
4	210	103	4	−	7	1	12	Unremarkable

*NPWTi-d* Negative Pressure Wound Therapy with instillation and dwelling, *SSI* Surgical site infection, *SBO* Small bowel obstruction

No SSI was observed postoperatively. One patient had postoperative ileus. The median postoperative hospital stay was 9 days (range: 7–14 days), the median number of days to confirm epithelialization was 11.5 days (range: 10–16 days), and the number of outpatient visits after leaving hospital was 0 or 1.

## Discussion

We found that closure using NPWTi-d and delayed primary closure was an effective therapy for stoma wound closure. There were no cases of SSI, and the burden on medical staff and patients during hospitalization was, therefore, decreased. In addition, the patients did not have to treat the wound themselves after discharge and had fewer outpatient visits.

A temporary stoma is commonly used when a low pelvic anastomosis is performed in rectal cancer and benign diseases. The most dreaded complication of a low pelvic anastomosis is an anastomotic leak; therefore, a temporary stoma is performed [8, 9].

SSI is an important and common complication after stoma closure. Peristomal skin reportedly harbors a considerable number of enteric bacteria, and since the procedure also entails enteric anastomosis, stoma closure is considered a clean-contaminated procedure [10]. Generally, risk factors for SSI include radiation therapy, chemotherapy, obesity, diabetes, long-term steroid administration, and immunosuppressant administration [11, 12]. SSI is associated with an increased burden of treatment for medical staff and patients. It causes prolonged postoperative hospital stay, increase in outpatient visits, additional home health care utilization, and increased medical costs. Additionally, as a late complication of SSI after stoma closure, an abdominal incisional hernia may develop. An abdominal incisional hernia can significantly reduce a patient’s quality of life and may require re-operation. It therefore leads to a further increase in medical costs.

Other steps have been taken to reduce SSI following stoma closure. With primary suture closure, the wound is closed immediately, and a dead space is formed. The subcutaneous fluids cannot drain, and an abscess may form. There are some reports of adding a drainage tube to the subcutaneous layer under the wound; however, this also has a high infection rate of about 20% [11].

Purse-string suturing has one of the lowest infection rates, and its usefulness has been demonstrated [1, 4, 11]. However, this method can take up to 30 days for granulation and epithelialization [13]. Continuous wound care and outpatient visits are required until the wound is healed, and it may be difficult for elderly patients to perform self-care procedures such as cleaning the wound. NPWT therapy is thought to promote granulation and wound healing. It increases wound blood flow, promotes granulation tissue formation, reduces edema, and removes exudate and inactive tissue by sealing the wound and applying negative pressure drainage. Currently, although NPWT is used for various surgical cases, its prophylactic use is still not considered vital in digestive surgery. A study on the use of NPWT in the prevention of SSI and shortening of wound healing period after stoma closure was not able to show the efficacy of NPWT compared to that of purse-string sutures in significantly reducing the wound healing time [5]. Additionally, local infection may occur as an adverse event with NPWT [5, 14].

NPWTi-d can prevent bacterial growth by automatic cleansing of the wound surface and removal of dissolving devitalized tissue and exudate early and aggressively. By using NPWTi-d on the stoma closure wound, the promotion of granulation may reduce the dead space and the risk of SSI [15–21]. It may also shorten the wound healing period. Additionally, delayed primary closure may further shorten the epithelialization time. This will reduce the burden on medical staff and patients and lead to a reduction in inpatient duration and outpatient visits.

This study has several limitations. First, this study had a very small sample size. Second, this was a non-controlled retrospective cohort study. The future study should be comparing the effects of NPWTi-d with those of purse string closure or primary closure. Third, it was not verified for cost-effectiveness. The cost-effectiveness should be assessed of NPWTi-d for preventing surgical site infection.

## Conclusions

Combined treatment with NPWTi-d and delayed primary closure of stoma closure wounds may be a new wound management technique, which can reduce the risk of SSI and shorten the wound healing period. In the future, it is desirable to study with a larger sample size.

## Data Availability

Not applicable.

## Abbreviations

SSI: Surgical site Infection
NPWTi-d: Negative pressure wound therapy with instillation and dwelling
NPWT: Negative pressure wound therapy
POD: Postoperative day
BMI: Body Mass Index
